# Magnetic Nanoparticle-Based High-Performance Positive and Negative Magnetic Resonance Imaging Contrast Agents

**DOI:** 10.3390/pharmaceutics15061745

**Published:** 2023-06-15

**Authors:** Tirusew Tegafaw, Shuwen Liu, Mohammad Yaseen Ahmad, Abdullah Khamis Ali Al Saidi, Dejun Zhao, Ying Liu, Sung-Wook Nam, Yongmin Chang, Gang Ho Lee

**Affiliations:** 1 Department of Chemistry, College of Natural Sciences, Kyungpook National University, Taegu 41566, Republic of Korea; tegafawtirusew@yahoo.com (T.T.); liushuwen0701@gmail.com (S.L.); yaseen.knu@gmail.com (M.Y.A.); abdullah_al_saidi@hotmail.com (A.K.A.A.S.); djzhao.chem@gmail.com (D.Z.); ly1124161@gmail.com (Y.L.); 2 Department of Molecular Medicine, School of Medicine, Kyungpook National University, Taegu 41944, Republic of Korea; nams@knu.ac.kr

**Keywords:** magnetic nanoparticle, magnetic resonance imaging, contrast agent, high-performance

## Abstract

In recent decades, magnetic nanoparticles (MNPs) have attracted considerable research interest as versatile substances for various biomedical applications, particularly as contrast agents in magnetic resonance imaging (MRI). Depending on their composition and particle size, most MNPs are either paramagnetic or superparamagnetic. The unique, advanced magnetic properties of MNPs, such as appreciable paramagnetic or strong superparamagnetic moments at room temperature, along with their large surface area, easy surface functionalization, and the ability to offer stronger contrast enhancements in MRI, make them superior to molecular MRI contrast agents. As a result, MNPs are promising candidates for various diagnostic and therapeutic applications. They can function as either positive (T_1_) or negative (T_2_) MRI contrast agents, producing brighter or darker MR images, respectively. In addition, they can function as dual-modal T_1_ and T_2_ MRI contrast agents, producing either brighter or darker MR images, depending on the operational mode. It is essential that the MNPs are grafted with hydrophilic and biocompatible ligands to maintain their nontoxicity and colloidal stability in aqueous media. The colloidal stability of MNPs is critical in order to achieve a high-performance MRI function. Most of the MNP-based MRI contrast agents reported in the literature are still in the developmental stage. With continuous progress being made in the detailed scientific research on them, their use in clinical settings may be realized in the future. In this study, we present an overview of the recent developments in the various types of MNP-based MRI contrast agents and their in vivo applications.

## 1. Introduction

Magnetic resonance imaging (MRI) is a widely used, advanced and effective diagnostic imaging technique owing to its distinct advantages over other imaging modalities [1,2,3,4]. Its unique features include high spatial resolution [5,6], excellent soft-tissue contrast [7,8,9], lack of ionizing radiation risk [10,11], and high imaging depths. Owing to these factors, MRI is often the preferred choice for diagnosing various medical conditions, particularly in pregnant women and children who may be sensitive to ionizing radiation [10,12,13]. This is because MRI is operated under low-energy radiofrequency radiation in contrast to invasive X-ray computed tomography (CT) and positron emission tomography (PET) scans which use high-energy ionizing radiation and can damage cells and tissues, causing side effects. Although MRI supports high spatial resolution (0.05–0.5 mm) [5], its sensitivity is relatively low [5] because of the inherently small population difference between two proton spin energy states in nuclear magnetic resonance, making the detection of small biological events, such as early-stage small lesions, difficult. However, MRI sensitivity can be improved using MRI contrast agents because they accelerate proton spin relaxation and enhance proton signals [14,15]. Various MRI contrast agents have been investigated and developed to achieve this goal [16,17,18,19,20]. Importantly, their accumulation in the region of interest (ROI) compared to surrounding tissues generates image contrast between the ROI and surrounding tissues, allowing for sensitive detection and diagnosis of the ROI.

MRI contrast agents do not produce signals but rather enhance the sensitivity and resolution of MRI via MR signal and contrast enhancements. Preclinical and clinical studies have investigated various types of MRI contrast agents [16,17,18,19,20], including Gd chelates such as Magnevist, Dotarem, Omniscan, and ProHance [21,22], which are currently being clinically developed as T_1_ MRI contrast agents. Other agents that have been extensively investigated include Mn chelates, Gd-based nanoparticles (NPs), Mn-based NPs, and superparamagnetic iron oxide nanoparticles (SPIONs). In addition, paramagnetic lanthanide (Ln)-based NPs (Ln = Dy, Ho, and Tb) have drawn interest as potential T_2_ MRI contrast agents in high MR fields owing to their appreciable paramagnetic moments at room temperature [23,24,25,26,27,28]. Molecular MRI contrast agents such as Gd chelates and Mn chelates typically have low longitudinal (r_1_) and transverse (r_2_) proton spin relaxivity and short blood circulation times owing to their rapid renal excretion, thus necessitating large amounts of injection doses to achieve their detection level. These requirements can increase the risk of biotoxicity because of the potential liberation of free metal ions in the body. For instance, free Gd^3+^ ions can cause nephrogenic systemic fibrosis (NSF) in patients with kidney diseases, resulting in the thickening and darkening of the skin and the reduction of organ function in the heart and lungs [22,29,30]. In addition, recent studies indicated that clinically developed Gd chelates could be deposited in the brain after repeated use and cause neurotoxicity [31,32]. SPION-based MRI contrast agents are more biocompatible than other metal-based NP contrast agents because iron is consumed in the human body as an essential element [33]; for example, iron is a central element in hemoglobin for oxygen binding. Among MNPs, only Fe-based NPs have been commercialized. In the past, Feridex, Sinerem, and Resovist, which are dextran-coated SPIONs, were approved by the Food and Drug Administration (FDA), USA for clinical trials in liver (Feridex and Resovist) and lymph node (Sinerem) imaging via intravenous injection [34,35,36,37,38]. In addition, Lumirem was approved by the FDA for gastrointestinal imaging via oral administration [34]. However, safety concerns led to Feridex’s and Sinerem’s withdrawal from the market because of back pain [36]. Lumirem was withdrawn from the market due to little use, but not for safety concerns [34]. Currently, only Resovist is allowed for liver imaging in limited countries [36,37,38].

Compared with metal chelates, magnetic NP (MNP)-based MRI contrast agents have numerous advantages. Not only do they have stronger magnetic moments and longer blood circulation times, resulting in higher contrast in images, but also support multifunctional applications such as multimodal imaging and the therapy of diseases via surface functionalization and drug delivery [39]. These advantages make them superior to molecular agents. Among the MNPs studied, SPIONs and Gd-based NPs have been most intensively investigated over the past decade owing to their outstanding magnetic properties over other MNPs. Notably, Gd-based NPs have higher r_1_ values than commercial Gd chelates [40,41,42,43,44,45]. Recently, researchers have explored paramagnetic NPs made of Dy, Ho, and Tb as potential alternatives to SPIONs as T_2_ MRI contrast agents at high MR fields (>3 T) because of their high r_2_ values. As the paramagnetic moments of these NPs increase with increasing MR field, they become comparable to those of SPIONs [23,24,25,26,27,28].

MNPs are composed of two parts: a magnetic core that enhances MR signals and a surface-coating ligand layer that ensures colloidal stability and nontoxicity. High colloidal stability in aqueous media is necessary for obtaining high-performance MRI function and maximal MR signal enhancement. Otherwise, the precipitation of MNPs can result in a reduced interaction between MNPs and water proton spins, resulting in reduced MR signal enhancement. In this review, we focused on newly developed, MNP-based MRI contrast agents and their in vivo applications. The review includes MNPs made of Gd, Mn, Fe, Dy, Ho, and Tb, which function as T_1_ MRI contrast agents; T_2_ MRI contrast agents; or dual-modal T_1_ and T_2_ MRI contrast agents. 

## 2. The Principle of Imaging Mode (T_1_ or T_2_ or Both)

**Scheme 1:**
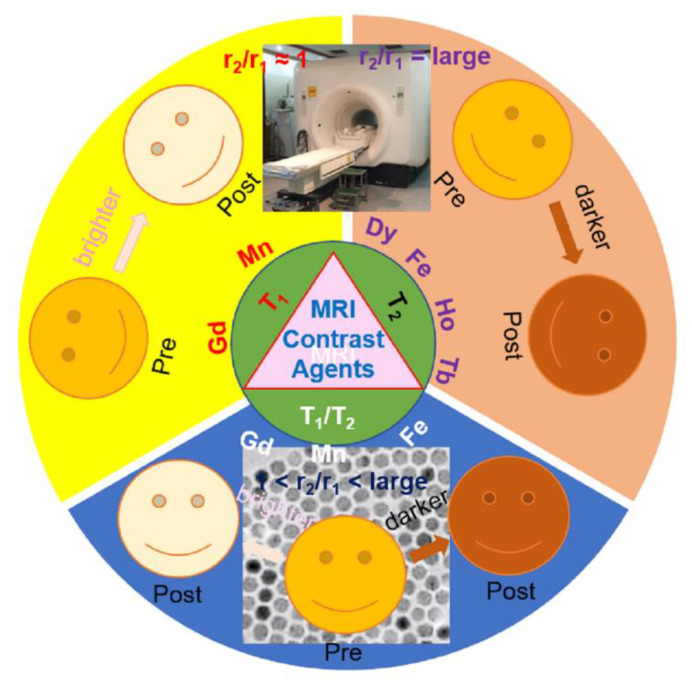
Types of imaging modes possible with MNPs made of Gd, Mn, Fe, Dy, Ho, and Tb.

The effectiveness of MNPs as MRI contrast agents depends on their r_1_ and r_2_ values, and their r_2_/r_1_ ratios [46,47]. As the longitudinal (T_1_) proton spin relaxation always occurs with the transverse (T_2_) proton spin relaxation, but not the other way around, the ideal T_1_ MRI contrast agent should have high r_1_ values and r_2_/r_1_ ratios close to 1.0. In contrast, the ideal T_2_ MRI contrast agent should have high r_2_ values and high r_2_/r_1_ ratios to solely induce T_2_ proton spin relaxation [46,47]. Dual-modal T_1_ and T_2_ MRI contrast agents should have both high r_1_ and r_2_ values, with r_2_/r_1_ ratios between those of ideal T_1_ and ideal T_2_ MRI contrast agents. The primary parameter that affects r_1_ and r_2_ values and r_2_/r_1_ ratios is the MNP composition. The particle size and surface-coating ligand are also important. The types of imaging modes possible with MNPs are summarized in Table 1 and Scheme 1.

### 2.1. T_1_ Imaging Mode

The longitudinal (T_1_) proton spin relaxation primarily depends on metal ion spin layers present on the MNP surfaces, which interact with nearby proton spins, accelerating T_1_ proton spin relaxation along the z-axis (the so-called inner sphere model [48,49,50]). Therefore, MNPs made of Gd^3+^ (s = 7/2), Mn^2+^ (s = 5/2), and Fe^3+^ (s = 5/2) can contribute to T_1_ proton spin relaxation because their slow s-electrons closely match with slow proton spin relaxation [48]. Gd^3+^ strongly induces T_1_ proton spin relaxation because of its highest electron spin magnetic moment (s = 7/2) among the periodic table elements [51]. Conversely, fast electrons with orbital angular moment components do not match with slow proton spin relaxation and thus negligibly induce T_1_ proton spin relaxation [48], resulting in negligible r_1_ values. For example, MNPs made of Ln^3+^ (Ln = Dy, Ho, and Tb) negligibly induce T_1_ proton spin relaxation because of their 4f-electrons with orbital angular moment components [23,24,25,26,27,28].

Recent studies indicate that the particle diameter of Fe-based NPs is critical in achieving optimal T_1_ proton spin relaxation because smaller NPs offer greater surface exposure of Fe^3+^ to the surrounding water protons [52,53,54,55,56,57,58]. Park and co-workers reported that the optimal particle diameter range of Gd-based NPs for maximal T_1_ proton spin relaxation or the maximal r_1_ value is 1–2.5 nm [40]. They proposed the cooperative effects of surface Gd^3+^ in accelerating T_1_ proton spin relaxation to explain this.

### 2.2. T_2_ Imaging Mode

MNPs become magnetized upon exposure to an external magnetic field, resulting in local magnetic field fluctuations around the MNPs because of their thermal motion in solution [59,60,61]. The local magnetic field fluctuations accelerate the transverse (T_2_) proton spin relaxation (so-called outer sphere model [48,49,50]), which corresponds to the dephasing of proton spin magnetic moments along the xy plane. MNPs with high magnetic moments, such as SPIONs, can generate high local magnetic field fluctuations that induce strong T_2_ proton spin relaxation, resulting in high r_2_ values. Note that MNPs made of Dy, Ho, and Tb exhibit appreciable paramagnetic moments at room temperature and thus exhibit appreciable r_2_ values [23,24,25,26,27,28,62,63,64]. They solely induce T_2_ proton spin relaxation, resulting in negligible r_1_ values. Notably, these paramagnetic moments increase with increasing MR field, resulting in even higher r_2_ values than those of SPIONs at very high MR fields [62].

### 2.3. T_1_ and T_2_ Dual-Imaging Mode

Conventional MRI contrast agents can serve as either T_1_ or T_2_ MRI contrast agents. However, the introduction of T_1_ and T_2_ dual-modal MRI contrast agents has enabled more accurate diagnosis of disease via complementary T_1_ and T_2_ MR images [65,66,67,68,69,70,71]. Dual-modal MR images can be easily obtained by changing the operational mode of the same MRI scanner, unlike other dual-modal imaging techniques (such as MRI-CT, CT-PET, and MRI-PET) that require a combination of two different imaging machines that are expensive, inconvenient, and time-consuming.

Designing dual-modal MNPs requires careful synthetic strategies to ensure they function in a dual-mode. The composition, particle diameter, and surface-coating ligand of MNPs can be controlled to optimize the r_1_ and r_2_ values [65,72,73,74,75,76]. For instance, small and hydrophilic surface-coating ligands can attract water molecules close to the MNPs, resulting in high r_1_ and r_2_ values, whereas large and hydrophobic surface-coating ligands do not, resulting in small r_1_ and r_2_ values [72,73,76]. Larger MNPs can provide higher r_2_ values and higher r_2_/r_1_ ratios owing to their enhanced magnetic moments and reduced amounts of surface metal ions and thus are more suitable as T_2_ MRI contrast agents [74]. The composition of MNPs may be controlled to obtain r_1_ and r_2_ values suitable for dual-modal imaging [65].

## 3. Examples of MNP-Based MRI Contrast Agents

### 3.1. T_1_ MRI Contrast Agents

#### 3.1.1. Gd-Based NPs

Among MNPs, Gd-based NPs possess the most suitable relaxivity properties for T_1_ MRI contrast agents, owing to Gd^3+^ having the highest spin magnetic moment (s = 7/2) among the elements in the periodic table. The r_1_ values of Gd-based NPs are higher than those [48,50] of Gd chelates because of the high density of Gd^3+^ per NP, making them high-performance T_1_ MRI contrast agents. Among various types of Gd-based NPs, such as Gd_2_O_3_, GdF_3_, and NaGdF_4_ NPs, the Gd_2_O_3_ NPs have been most intensively investigated thus far.

**Figure 1:**
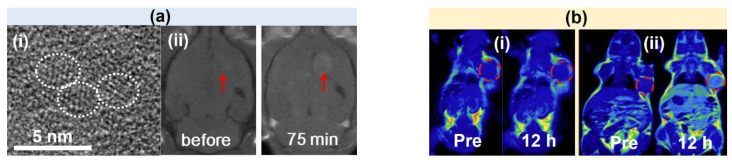
(**a**) (**i**) TEM image of D-glucuronic acid-coated Gd_2_O_3_ NPs (labeled as dotted circles) and (**ii**) in vivo T_1_ MR images of mouse brain tumor (labeled with arrows) before and 75 min after intravenous injection at 1.5 T [40]. (**b**) In vivo T_1_ MR images of mice bearing renal carcinoma tumor (labeled with red circles), before (labeled as “Pre”) and 12 h after intravenous injection of (**i**) Magnevist and (**ii**) PEG-Gd_2_O_3_ NPs at 3 T [78].

Park et al. synthesized D-glucuronic acid-coated Gd_2_O_3_ NPs (mean particle diameter = 1 nm) in polyol solvent [40]. The transmission electron microscope (TEM) image is presented in Figure 1a(i). The synthesized NPs exhibited a high r_1_ value of 9.9 s^−1^mM^−1^ and thus high positive T_1_ contrasts in mouse brain tumors after intravenous injection at 1.5 T (Figure 1a(ii)).

Recently, Yang et al. reported polyvinylpyrrolidone (PVP)-coated Gd_2_O_3_ NPs (PVP Mw = 10,000 amu and mean particle diameter = 2.5 ± 0.8 nm) with a high r_1_ value of 10.8 mM^−1^s^−1^ at 3 T [77]. Appreciable contrasts in T_1_ MR images of the tumor, kidney, bladder, and liver were observed after intravenous injection at 3 T.

Dai et al. reported a comparison study between polyethylene glycol (PEG)-coated (or PEGylated)-Gd_2_O_3_ NPs (PEG Mw = 600 amu and mean hydrodynamic diameter = 36.35 nm) and commercially available Magnevist (or gadopentetic acid) [78]. The PEGylated-Gd_2_O_3_ NPs exhibited a higher r_1_ value of 29.0 mM^−1^s^−1^ than that (= 4.2 mM^−1^s^−1^) of Magnevist at 3 T. The T_1_ MR images of tumor-bearing mice were obtained 3 T (Figure 1b). The contrast enhancement of PEGylated-Gd_2_O_3_ NPs in the tumor was stronger than that of Magnevist at the same injection dose, confirming the superiority of PEGylated-Gd_2_O_3_ NPs, compared with Magnevist.

#### 3.1.2. Mn-Based NPs

Mn-based NPs have emerged as alternatives to Gd-based NPs because of their lower toxicity. Mn-based NPs have shown appreciable paramagnetic moments at room temperature owing to Mn^2+^ (s = 5/2), but their paramagnetic moments are lower than those of Gd-based NPs because Mn^2+^ (s = 5/2) has a lower spin magnetic moment than Gd^3+^ (s = 7/2).

**Figure 2:**
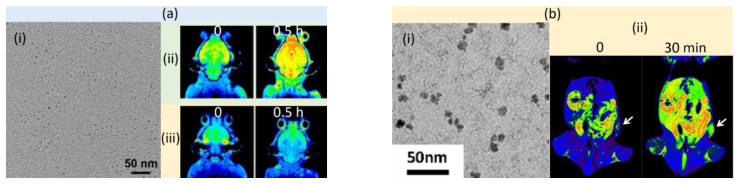
(**a**) (**i**) TEM image of USMnO@ZDS NPs and in vivo T_1_ MR images of mouse brains, before (labeled as “0”) and 0.5 h after intravenous injection of (**ii**) USMnO@ZDS and (**iii**) USMnO@DMSA at 9.4 T [79]. (**b**) (**i**) TEM image of uncoated Mn_3_O_4_ NPs and (**ii**) T_1_ MR images of Balb/c nude mice bearing a nasopharyngeal carcinoma xenografted tumor (indicated with white arrows), before (labeled as “0”) and 30 min after intravenous injection of Mn_3_O_4_ NPs at 3 T [81].

Wei et al. synthesized zwitterionic dopamine sulfonate (ZDS)-coated ultrasmall MnO NPs (simply, USMnO@ZDS) (mean particle diameter = 1.1 nm), which exhibited a high r_1_ value of 15.6 ± 0.4 mM^−1^s^−1^ and a high r_2_ value of 26.9 ± 1.1 mM^−1^s^−1^ at 0.5 T [79]. The TEM image of USMnO@ZDS is presented in Figure 2a(i). The USMnO@ZDS exhibited positive contrast enhancements in mouse brains after intravenous injection at 9.4 T, while *meso*-2,3-dimercaptosuccinic acid (DMSA)-grafted MnO NPs (simply, USMnO@DMSA) did not (Figure 2a(ii)). This indicated that the surface modification of MnO NPs with proper ligands such as ZDS, not DMSA, is crucial for the penetration of the NPs through the brain–blood barrier.

Li et al. synthesized PEG-coated MnO NPs (PEG Mw = 600 amu and mean particle diameter = 1.9 nm), with a high r_1_ value of 12.942 s^−1^mM^−1^ and an r_2_/r_1_ ratio of 4.66 at 3 T [80]. They conjugated the PEG-MnO NPs with AS1411 aptamer to target tumors, resulting in AS1411-PEG-MnO NPs that retained contrast in the tumor for up to 7 days after intravenous injection. However, the contrast provided by PEG-MnO NPs disappeared after 1 day of injection because of their lack of tumor targeting.

Xiao et al. prepared ligand-free Mn_3_O_4_ NPs (mean particle diameter = 9 nm) using laser ablation in aqueous media [81], as shown in a TEM image presented in Figure 2b(i). These uncoated Mn_3_O_4_ NPs had an r_1_ value of 8.26 mM^−1^s^−1^ at 3 T, which did not significantly affect the cell viability, owing to their low toxicity. T_1_ MR images at 3 T revealed high positive contrast enhancements in the xenografted tumor 30 min after intravenous injection (Figure 2b(ii)), demonstrating their potential as a T_1_ MRI contrast agent.

### 3.2. T_2_ MRI Contrast Agents

#### 3.2.1. Fe-Based NPs

Because of the high magnetic moments and better biocompatibility of Fe-based NPs compared with other metal-based NPs, they have been extensively investigated as T_2_ MRI contrast agents.

**Figure 3:**
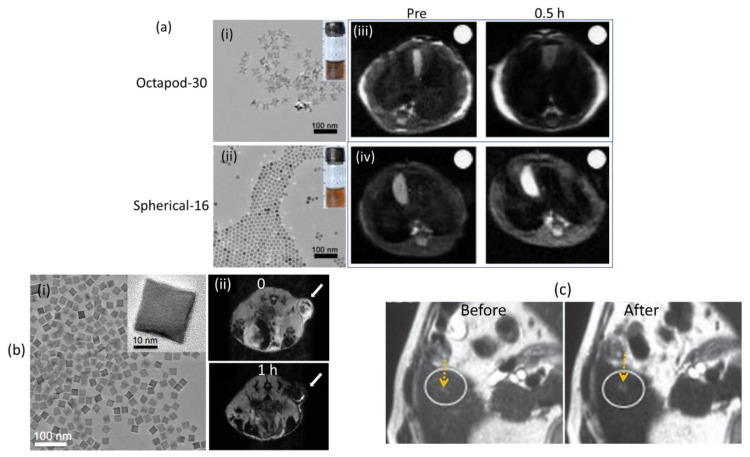
(**a**) TEM images of (**i**) octapod-30 and (**ii**) spherical-16 NPs (scale bars are 100 nm). T_2_ MR images in mouse livers at 7 T before (or Pre) and 0.5 h after intravenous injection of (**iii**) octapod-30 and (**iv**) spherical-16 NPs (the white dot is the signal reference using water in an NMR tube) [82]. (**b**) (**i**) TEM image of FIONs and (**ii**) in vivo T_2_ MR images of the tumor site at 3 T before (or 0) and 1 h after intravenous injection of WFIONs (arrows indicate the tumor sites) [85]. (**c**) A small (5 mm) hepatocellular carcinoma (indicated with arrows) proven by biopsy. T_2_ MR images at 1.5 T before and after intravenous injection of Resovist into patients showing clearer faint focal hyperintensity after injection in the hepatic segment [38].

Zhao et al. highlighted the importance of size and morphology tuning for the development of SPIONs with high r_2_ values [82]. Octapod NPs showed higher r_2_ values than spherical NPs, with r_2_ values of 679.25 ± 30, 209.03 ± 15, 125.86 ± 9, and 59.91 ± 6 30 mM^−1^s^−1^ at 7 T for octapod-30 (mean edge length = 30 nm), octapod-20 (mean edge length = 20 nm), spherical-16 (mean particle diameter = 16 nm), and spherical-10 NPs (mean particle diameter = 10 nm), respectively. The TEM images of octapod-30 and spherical-16 NPs are presented in Figure 3a(i,ii) respectively. In vivo results at 7 T demonstrated that octapod-30 NPs produced higher MR contrasts in the tumor than those of spherical-16 NPs (Figure 3a(iii,iv).

Wang et al. reported the one-pot synthesis of ultrasmall SPIONs (uBSPIOs) using bovine serum albumin as a scaffold [83]. The resulting uBSPIOs (mean particle diameter = 4.78 ± 0.55 nm) exhibited a high r_2_ value of 444.56 ± 8.82 mM^−1^s^−1^ at 7 T. These uBSPIOs provided high negative contrasts in the T_2_ MR images of the tumor. In addition, the uBSPIOs demonstrated no cytotoxicity in vitro and negligible organ toxicity in vivo.

Leal et al. synthesized PEGylated SPIONs (mean particle diameter = 6 nm) with PEG molecular weight (MW) ranging from 600 to 8000 amu [84]. The PEG3000-SPIONs (PEG Mw = 3000 amu) exhibited better in vivo performance, with longer circulation times and slower liver uptake than other MW PEG-coated SPIONs. This was because high MW PEGs (6000–8000 amu) led to NP aggregations, whereas low MW PEGs (≤1500 amu) could not stabilize the NPs in physiological media. The PEG1500-, PEG3000-, PEG6000- and PEG8000-SPIONs had r_2_ values of 90, 103, 190, and 180 mM^−1^s^−1^ at 1.5 T and the r_2_ values of 114, 151, 253, and 254 mM^−1^s^−1^ at 9.4 T, respectively. After intravenous injection into mice tails at 9.4 T, the PEGylated SPIONs exhibited negative contrast enhancements in T_2_ MR images, demonstrating their potential as T_2_ MRI contrast agents.

Lee et al. synthesized ferrimagnetic iron oxide nanocubes (FIONs) (mean edge length = 22 nm, as presented in a TEM image in Figure 3b(i)) and encapsulated them in PEG-phospholipids to obtain water-dispersible FIONs (WFIONs) [85]. The WFIONs exhibited an extremely high r_2_ value of 761 mM^−1^s^−1^ at 3 T, which is close to the theoretically predicted maximum r_2_ value of ~800 mM^−1^s^−1^ for 22 nm sized NPs with a saturation magnetization of 106 emu/g. Notably, their study demonstrated that FION aggregates could generate a very strong magnetic field that caused nearby water proton spins to completely lose their phase, leading to a decrease in their r_2_ value as the NP size increased. In vivo MR images revealed a distinct signal decrease in the tumor site 1 h after intravenous injection (Figure 3b(ii)).

Maurea et al. reported the clinical applications of a commercial T_2_ MRI contrast agent Resovist in humans [38]. Resovist is carboxydextrane-coated SPION with a hydrodynamic diameter ranging from 45 to 60 nm and r_2_ and r_1_ values of 151.0 and 25.4 mM^−1^s^−1^, respectively [35]. As shown in Figure 3c, hepatocellular carcinoma could be more clearly observed after intravenous injection compared with no injection (labeled as before) [38].

#### 3.2.2. Dy-Based NPs

Dy-based NPs have gained significant interest as a new class of T_2_ MRI contrast agents because of their appreciable paramagnetic moments at room temperature. Dy has the highest effective magnetic moment (μ_eff_) (10.65 μ_B_) among the elements in the periodic table [51]. Currently, most MRI contrast agents are operated at clinical MR fields (1.5–3 T). However, Dy-based NPs can become more powerful at higher MR fields such as 7 and 9.4 T because their paramagnetic moment increases with the increasing MR field. This will trigger a new opportunity to generate powerful T_2_ MRI contrast agents suitable for high-field MRI scanners. In addition to T_2_ relaxation by outer sphere model, Curie-spin relaxation is also important for Dy-based NPs [86,87]. This is because T_2_ relaxation is induced by the modulated magnetic dipolar interaction between proton spins and thermally averaged electronic spins (or Curie-spins) of Dy^3+^ in NPs by NP motion. This Curie-spin relaxation becomes important in high MR fields.

González-Mancebo et al. synthesized rhombus-like DyF_3_ NPs (mean length × width = 110 × 50 nm) in ethylene glycol, which served as the solvent and surface-coating ligand [88]. The DyF_3_ NPs exhibited a remarkably high r_2_ value of 380.4 mM^−1^s^−1^ at 9.4 T, with a high r_2_/r_1_ ratio of 559.37, demonstrating their potential as highly effective T_2_ MRI contrast agents.

Kattel et al. reported on the effectiveness of D-glucuronic acid-coated Dy_2_O_3_ NPs and Dy(OH)_3_ nanorods as T_2_ MRI contrast agents [89]. The D-glucuronic acid-coated Dy_2_O_3_ NPs (mean particle diameter = 3.2 nm) and Dy(OH)_3_ nanorods (mean diameter × length = 20 × 300 nm) exhibited negligible r_1_ values and high r_2_ values of 65.04 and 181.57 s^−1^mM^−1^ at 1.5 T, respectively. Dy_2_O_3_ NPs and Dy(OH)_3_ nanorods also produced clear negative contrast enhancements of T_2_ MR images of mouse liver and kidneys at 3 T after intravenous injection.

**Figure 4:**
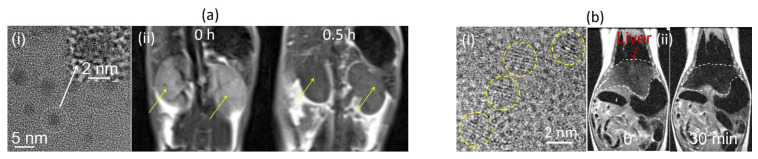
(**a**) (**i**) TEM image of amorphous carbon-coated Dy_2_O_3_ NPs and (**ii**) T_2_ MR images in mouse kidneys (labeled with arrows) before (or 0 h) and 0.5 h after intravenous injection of amorphous carbon-coated Dy_2_O_3_ NPs at 3 T [90]. (**b**) (**i**) TEM image of PAA-coated Dy_2_O_3_ NPs (labeled with yellow circles) and (**ii**) T_2_ MR images of mouse liver (labeled with dotted lines and an arrow) before (or 0) and 30 min after intravenous injection of PAA-coated Dy_2_O_3_ NPs into mice tails at 3 T [91].

Recently, Yue et al. synthesized hydrophilic, nearly nontoxic, amorphous carbon-coated Dy_2_O_3_ NPs (mean particle diameter = 3.0 nm, as presented in a TEM image in Figure 4a(i)) [90]. The carbon coating was achieved through dextrose polymerization on the Dy_2_O_3_ NP surfaces, which left hydroxyl groups on the NP surfaces and made the amorphous carbon-coated Dy_2_O_3_ NPs colloidally stable in aqueous media and nearly nontoxic. The amorphous carbon-coated NPs had r_1_ and r_2_ values of 0.1 and 5.7 s^−1^mM^−1^ at 3 T, respectively, with an r_2_/r_1_ ratio of 57. In vivo T_2_ MR images of mouse kidneys at 3 T exhibited negative contrasts after intravenous injection, demonstrating their potential as T_2_ MRI contrast agents (Figure 4a(ii)). In addition, the NPs exhibited broad photofluorescence at 490 nm (400–600 nm) upon excitation at 370 nm due to the fluorescent nature of the amorphous carbon-coating layers on the NP surfaces. Therefore, amorphous carbon-coated Dy_2_O_3_ NPs are suitable as dual-modal T_2_ MRI-fluorescence imaging (FI) agents.

Marasini et al. developed colloidally stable poly(acrylic) acid (PAA)-coated Dy_2_O_3_ NPs (PAA Mw = 1800 amu and mean particle diameter = 1.7 nm, as presented in a TEM image in Figure 4b(i)) using a simple polyol synthesis [91]. The r_1_ value was negligible, but the r_2_ value increased with increasing MR field such that it was 2.01 s^−1^mM^−1^ at 3 T and 11.31 s^−1^mM^−1^ at 9.4 T. In vivo T_2_ MR images at 3 T exhibited clear negative contrast enhancements in mouse livers after intravenous injection (Figure 4b(ii)), demonstrating their potential as a T_2_ MRI contrast agent.

Recently, Gómez-González et al. synthesized PAA-coated DyPO_4_ NPs (PAA Mw = 1800 amu) with tunable particle diameters ranging from 23 to 57 nm using a homogeneous precipitation method in butanol [92]. They found that the r_2_ values were 395, 432, and 516 mM^−1^s^−1^ for 23, 37, and 57 nm NPs at 9.4 T, respectively. These high r_2_ values confirmed the suitability of PAA-coated DyPO_4_ NPs as T_2_ MRI contrast agents.

#### 3.2.3. Ho-Based NPs

Ho-based NPs exhibit appreciable paramagnetic moments at room temperature, similar to Dy-based NPs, owing to Ho with high μ_eff_ of 10.60 μ_B,_ the second highest value among elements in the periodic table [51]. Consequently, they are potential candidates for T_2_ MRI contrast agents at high MR fields similar to Dy-based NPs.

Gómez-González et al. synthesized cube-shaped HoPO_4_ NPs that were grafted with PAA (Mw = 1800 amu) to investigate the relationship between r_2_ value and MR field (1.44 and 9.4 T) and between r_2_ value and NP diameter (27, 48, and 80 nm) [93]. The r_2_ value of HoPO_4_@PAA NPs increased with increasing NP diameter at 1.44 T, but failed for the largest NPs at 9.4 T because of their aggregation; consequently, the 48 nm NPs exhibited the highest r_2_ value of 489.91 mm^−1^s^−1^ at 9.4 T. In vivo studies using 48 nm HoPO_4_@PAA NPs at 9.4 T revealed that, after intravenous injection, the NPs showed distinct T_2_ contrasts in both the liver and spleen, demonstrating their potential as T_2_ MRI contrast agents.

Atabaev et al. reported an r_2_ value of 23.47 mM^−1^s^−1^ at 1.5 T for PEG-coated Ho_2_O_3_ NPs (PEG Mn = 4000 amu) with a particle diameter of 67–81 nm [94], which is high enough to be used as T_2_ MRI contrast agents.

González-Mancebo et al. investigated the r_2_ values of HoF_3_ NPs at 9.4 T as a function of particle size and composition [88]. They synthesized two different types of HoF_3_ NPs in ethylene glycol, which served as the solvent and surface-coating ligand; ellipsoid-like HoF_3_ NPs (HoF-el) (mean length × width = 70 × 30 nm) and rhombus-like HoF_3_ NPs (HoF-rh) (mean length × width = 110 × 50 nm). The NPs exhibited r_2_ values of 349.98 mM^−1^s^−1^ and 608.4 mm^−1^s^−1^ at 9.4 T for HoF-el and HoF-rh, respectively. The increase in r_2_ value with increasing particle size was attributed to the higher magnetization of larger NPs than smaller ones. The high r_2_ values indicated that the synthesized NPs should be useful as high-field T_2_ MRI contrast agents.

Recently, Zhang et al. conducted a study on PEG-HoF_3_ NPs (PEG Mw = 4000 amu and mean particle diameter = 38 nm) as a T_2_ MRI contrast agent for cancer diagnosis [95]. The NPs had an r_2_ value of 117.51 mM^−1^s^−1^ at 7 T, and 24 h after intravenous injection, negative (or darker) contrasts in T_2_ MR images in the tumors of tumor-bearing mice were observed because of the accumulation of NPs in the tumor, demonstrating the potential of the NPs as a T_2_ MRI contrast agent.

**Figure 5:**
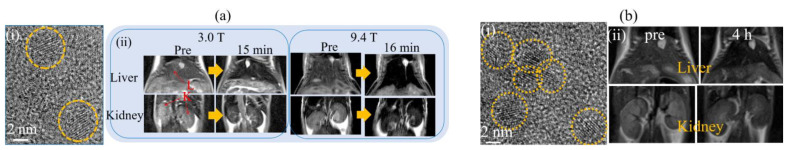
(**a**) (**i**) TEM image of PAA-coated Ho_2_O_3_ NPs (labeled with dotted circles) and (**ii**) in vivo T_2_ MR images of mouse liver (labeled as “L”) and kidneys (labeled as “K”), before (labeled as “Pre”) and 15 or 16 min after intravenous injection of PAA-coated Ho_2_O_3_ NPs into mice tails at 3.0 and 9.4 T [96]. (**b**) (**i**) TEM image (labeled with dotted circles) and (**ii**) in vivo T_2_ MR images of mouse liver and kidneys at 3 T, before (labeled as “pre”) and 4 h after intravenous injection of PEGD250-coated Ho_2_O_3_ NPs [98].

Marasini et al. synthesized PAA-coated Ho_2_O_3_ NPs (PAA Mw = 1800 amu and mean particle diameter = 1.7 nm) using one-pot polyol synthesis [96]. The TEM image is presented in Figure 5a(i). The NPs were nearly nontoxic and colloidally stable in aqueous media without precipitation after synthesis because of their hydrophilic and biocompatible PAA coating. The PAA-coated Ho_2_O_3_ NPs exhibited an appreciable r_2_ value of 1.44 s^−1^mM^−1^ at 3 T and an enhanced r_2_ value of 9.20 s^−1^mM^−1^ at 9.4 T. In vivo T_2_ MR images of the liver and kidneys exhibited strong negative contrast enhancements at 3 T, and stronger negative contrast enhancements at 9.4 T, confirming the effectiveness of the NPs as T_2_ MRI contrast agents at high-MR fields (Figure 5a(ii)).

Liu et al. synthesized polyethylenimine (PEI)-coated Ho_2_O_3_ NPs using a one pot polyol method (PEI Mn = 1200 and 60,000 amu) and investigated their potential as T_2_ MRI contrast agents [97]. The NPs exhibited mean particle diameters of 2.05 and 1.90 nm and appreciable r_2_ values of 13.1 and 9.9 s^−1^mM^−1^ for the PEI1200 and PEI60000-coated NPs, respectively. The same authors synthesized Ho_2_O_3_ NPs grafted with PEG diacid (PEGD)250 (Mw = 250 amu), PEGD600 (Mw = 600 amu), and PAA1800 (Mw = 1800 amu) using the one-pot polyol method (mean particle diameter = 2.1, 2.1, and 1.7 nm, respectively) [98]. The r_2_ value decreased with increasing ligand-size such that 30.39 s^−1^mM^−1^ (PEGD250) < 11.33 s^−1^mM^−1^ (PEGD600) < 1.44 s^−1^mM^−1^ (PAA1800). In vivo T_2_ MRI studies were performed at 3 T using PEGD250-coated Ho_2_O_3_ NPs because they had the highest r_2_ value among the samples. The TEM image of PEGD250-coated Ho_2_O_3_ NPs is presented in Figure 5b(i), and appreciable negative contrast enhancements in the liver and kidneys were observed (Figure 5b(ii)), demonstrating their potential as T_2_ MRI contrast agents.

#### 3.2.4. Tb-Based NPs

Tb-based NPs have shown great potential as T_2_ MRI contrast agents at high MR fields owing to their appreciable paramagnetic moments at room temperature, which were similar to Dy- and Ho-based NPs because Tb has μ_eff_ of 9.72 μ_B_ [51]. Despite their promising potential as T_2_ MRI contrast agents at high MR fields, only a few studies on Tb-based NPs have been reported.

**Figure 6:**
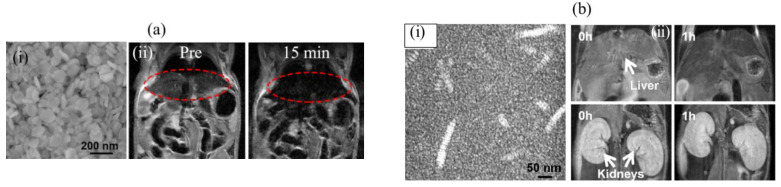
(**a**) (**i**) TEM image of PEI-coated TbF_3_ NPs and (**ii**) in vivo T_2_ MR images of Kunming mouse liver (labeled as dotted circles), before (labeled as “Pre”) and 15 min after intravenous injection of PEI-coated TbF_3_ NPs at 7 T [99]. (**b**) (**i**) STEM image of PEG-TbNRs and (**ii**) in vivo T_2_ MR images of mouse liver and kidneys (labeled with arrows), before (labeled as “0 h”) and 1 h after intravenous injection of the PEG-TbNRs at 9.4 T [24].

Zheng et al. synthesized PEI-coated TbF_3_ NPs (PEI Mn = 25,000 amu) using a facile solvothermal method [99]. The NPs had a plate morphology (mean particle diameter × thickness = 160 × 29 nm), as presented in the TEM image in Figure 6a(i). They obtained high r_2_ values of 6.54 and 395.77 mM^−1^s^−1^ at 0.5 and 7 T, respectively. An in vivo T_2_ MRI study at 7 T revealed that after injection into mouse tail veins, the MR signal intensities in the liver, spleen, and kidneys decreased significantly after injection, indicating the accumulation of NPs in these organs. An example of a liver MR image 15 min after injection is presented in Figure 6a(ii).

Marasini et al. synthesized D-glucuronic acid-coated Tb_2_O_3_ NPs (mean particle diameter = 2.0 nm) as a potential dual-modal T_2_ MRI-FI agent because Tb also emits photons in the 545 nm region [100]. The D-glucuronic acid-coated NPs exhibited r_2_ values of 7.68 mM^−1^s^−1^ at 1.5 T, 33.97 mM^−1^s^−1^ at 3 T, and 53.67 mM^−1^s^−1^ at 9.4 T, indicating that they have r_2_ values suitable as T_2_ MRI contrast agents. Furthermore, the NPs exhibited fluorescence in the green region, making them suitable as dual-modal T_2_ MRI-FI agents.

Recently, Caro et al. investigated the potential of PEG-coated Tb-based nanorods (PEG-TbNRs) as multimodal bioimaging agents [24]. The PEG-TbNRs (PEG Mw = 3000 amu and mean particle diameter × length = 2 × 9 nm) exhibited high colloidal stability and excellent luminescent, magnetic, and X-ray attenuation properties. The scanning TEM (STEM) image demonstrating the nanorod morphology is presented in Figure 6b(i). The r_2_ values of PEG−TbNRs at 1.44 and 9.4 T were estimated to be 10.4 and 48.5 mM^−1^s^−1^, respectively. In vivo T_2_ MR images at 9.4 T exhibited appreciable negative contrast enhancements in the liver and kidneys after intravenous injection (Figure 6b(ii)), demonstrating the potential of PEG-TbNRs as T_2_ MRI contrast agents.

### 3.3. T_1_ and T_2_ Dual-Modal MNPs

Despite the promising potential of dual-modal imaging compared to single-modal imaging, there are limited reports on T_1_ and T_2_ dual-modal contrast agents in MRI [65,66,67,68,69,70,71,101,102,103,104,105,106,107,108,109,110,111,112]. MNPs used for dual-modal operation should have high r_1_ and r_2_ values with r_2_/r_1_ ratios higher than 1, but not excessively high. To use Gd-based NPs as T_1_ and T_2_ dual-modal contrast agents, the r_2_/r_1_ ratios should be increased because their ratios are close to 1, while the r_2_/r_1_ ratios of SPIONs should be decreased because their ratios are very high. Mn-based NPs can be used as T_1_ and T_2_ dual-modal contrast agents because of their suitable r_2_/r_1_ ratios. However, Ln-based NPs (Ln = Dy, Ho, and Tb) can only be used as T_2_ MRI contrast agents because they have negligible r_1_ values [96,97,98,99,100].

**Figure 7:**
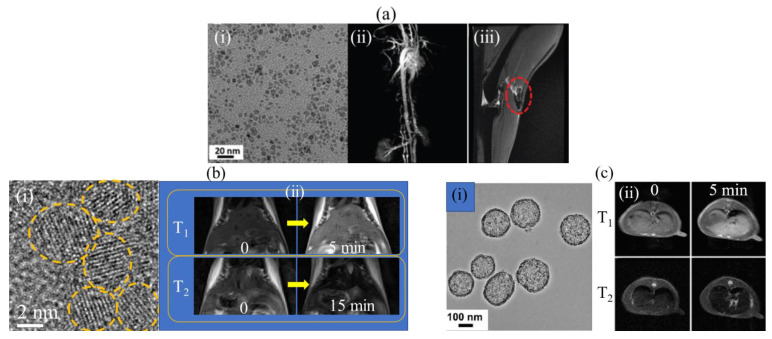
(**a**) (**i**) TEM image of PAA-coated Fe_3_O_4_ NPs (labeled with yellow circles), (**ii**) T_1_ MR images of rabbit vasculature after intravenous injection, and (**iii**) T_2_ MRI images of rabbit popliteal lymph node (dotted circle) after intravenous injection at 3 T [109]. (**b**) (**i**) TEM image of PASA-coated Gd_2_O_3_ NPs and (**ii**) in vivo T_1_ and T_2_ MR images of mouse livers, before (labeled as “0”) and 5 and 15 min after intravenous injection of PASA-coated Gd_2_O_3_ NPs into mice tails at 3 T [111]. (**c**) (**i**) TEM image of Mn-DMSSs and (**ii**) in vivo T_1_ and T_2_ MR images of rat livers, before (labeled as “0”) and 5 min after intravenous injection at 3 T [112].

Miao et al. synthesized PAA-coated Fe_3_O_4_ NPs (PAA = 1000 amu and mean particle diameter = 5.1 nm). The NPs exhibited r_1_ and r_2_ values of 10.52 and 38.97 mM^−1^s^−1^ (r_2_/r_1_ = 3.70) at 1.41 T, respectively [109]. The TEM image is presented in Figure 7a(i). The performance of the NPs as a dual-modal T_1_ and T_2_ MRI contrast agent was demonstrated from T_1_ and T_2_ MR images at 3 T, where positive contrasts were clearly observed in the rabbit vasculature (Figure 7a(ii)) and negative contrasts were clearly observed in the rabbit popliteal lymph node (dotted circle) (Figure 7a(iii)).

Li et al. developed monodispersed water-soluble and biocompatible ultrasmall magnetic iron oxide nanoparticles (mean particle diameter = 3.3 ± 0.5 nm) grafted with poly(methacrylic acid) (PMAC, M_n_ = 6359 amu) in aqueous media using a high-temperature coprecipitation method [104]. The PMAC-grafted NPs exhibited r_1_ = 8.3 and r_2_ = 35.1 s^−1^mM^−1^ (r_2_/r_1_ = 4.2) at 4.7 T, and demonstrated their potential as dual-modal T_1_ and T_2_ MRI contrast agents. After intravenous injection, positive and negative contrasts were observed in the T_1_ and T_2_ MR images of mice liver and kidneys, respectively.

Mekuria et al. reported on the encapsulation of Gd_2_O_3_ NPs (diameter = 3–5 nm) with 4.5 generation (G4.5) polyamidoamine (PAMAM) dendrimers and then, conjugated them with PEG (Mp = 10,000 amu) to obtain PEG-G4.5-Gd_2_O_3_ NPs [110]. The PEG-G4.5-Gd_2_O_3_ NPs exhibited a high r_1_ value of 53.9 mM^−1^s^−1^ and a high r_2_ value of 182.81 mM^−1^s^−1^ (r_2_/r_1_ = 3.4) at 7 T. An in vivo T_1_ MRI study showed that the PEG-G4.5-Gd_2_O_3_ NPs significantly enhanced signals in mouse intestines and kidneys. In addition, the T_2_ MRI study demonstrated a darker contrast in the kidneys, demonstrating the potential of G4.5-Gd_2_O_3_-PEG NPs as a dual-modal T_1_ and T_2_ MRI contrast agent.

Recently, Marasini et al. developed a dual-modal T_1_ and T_2_ MRI contrast agent by coating Gd_2_O_3_ NPs (mean particle diameter = 2 nm) with polyaspartic acid (PASP) (Mw = ~9900 amu) using the one-pot polyol method [111]. The TEM image is presented in Figure 7b(i). The synthesized NPs exhibited high r_1_ and r_2_ values of 19.1 and 53.7 mM^−1^s^−1^ (r_2_/r_1_ = 2.8) at 3 T, respectively. After intravenous injection of PASP-coated Gd_2_O_3_ NPs into the mice tails, T_1_ and T_2_ contrasts were observed in the T_1_ and T_2_ MR images of the mouse livers at 3 T, respectively (Figure 7b(ii)). This result showed that dual-modal T_1_ and T_2_ MRI contrast agents prepared using Gd_2_O_3_ NPs could be obtained by choosing the appropriate hydrophilic polymers as surface-coating ligands, such as the PASP used in this study.

Another promising candidate for dual-modal T_1_ and T_2_ MRI contrast agents is Mn-based NPs. Niu et al. developed manganese oxide nanocluster-loaded (diameter < 2 nm) dual-mesoporous silica spheres (Mn-DMSS; diameter = 100–200 nm, as presented in the TEM image in Figure 7c(i)) [112]. Mn-DMSSs exhibited a high r_1_ value of 10.1 mM^−1^s^−1^ and a high r_2_ value of 169.7 mM^−1^s^−1^ (r_2_/r_1_ = 16.8) at 3 T. An in vivo experiment on rats at 3 T demonstrated that Mn-DMSSs exhibited a 29% signal enhancement in the liver under T_1_ imaging mode and a 28% signal decrease under T_2_ imaging mode (Figure 7c(ii)), demonstrating the potential of Mn-DMSSs as dual-modal T_1_ and T_2_ MRI contrast agents.

## 4. Colloidal Stability, Biocompatibility, and Renal Excretion

It is essential that MNPs are coated with hydrophilic and biocompatible ligands to maintain their nontoxicity and colloidal stability in aqueous media. The colloidal stability of MNPs is critical for achieving high-performance MRI function because precipitated NPs lessen or negligibly contribute to inducing proton spin relaxation.

Compared with MNPs made of Gd, Mn, Dy, Ho, and Tb, Fe-based NPs are more biocompatible because iron is consumed in the human body as an essential element [33]; for example, it is used in hemoglobin for oxygen binding. For this reason, several Fe-based MNPs as MRI contrast agents, such as Feridex, Sinerem, and Resovist, were commercialized with approval by the FDA, USA [34,35,36,37,38], over other metal-based MNPs.

It is critical that MNPs should be nontoxic for biomedical applications [113,114]. Because MRI contrast agents are generally intravenously injected, it is preferred that MNPs are excreted through the renal system rather than the hepatobiliary pathway because the hepatobiliary excretion is relatively slow and MNPs could decompose during the excretion process, which would be toxic to the body. For example, free Gd^3+^ ions liberated from Gd chelates into the body could cause NSF [22,29,30]. For renal excretion, MNPs should be ultrasmall with hydrodynamic diameters less than 5 nm [115,116,117] because the glomerular filtration diameter in the kidneys is 4.5–5 nm [115]. It is also essential that the kinetic stability of MNPs is high so that they do not decompose until they are excreted through the renal system as urine.

**Figure 8:**
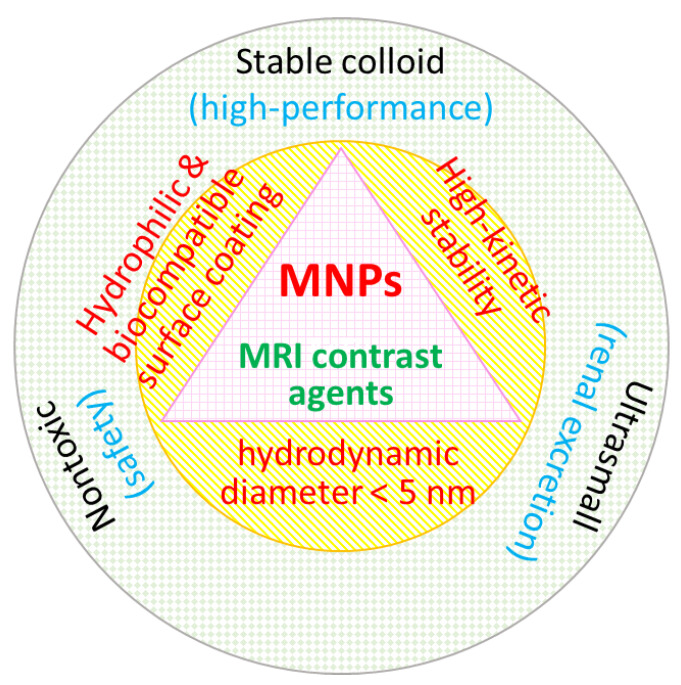
Conditions for MNPs for safe, in vivo application.

As summarized in Figure 8, MNPs as MRI contrast agents for safe, in vivo applications should be kinetically stable (i.e., no decomposition), coated with hydrophilic and biocompatible polymers for nontoxicity and colloidal stability, and ultrasmall with hydrodynamic diameters less than 5 nm for renal excretion. Under these conditions, MNPs can serve as high-performance MRI contrast agents which are superior to commercial molecular MRI contrast agents. In particular, the magnetic properties of MNPs made of Gd, Dy, Ho, and Tb are nearly size independent and thus can be made ultrasmall for renal excretion. They can strongly induce proton spin relaxation at high MR fields, implying that those MNPs are potential candidates for a new type of MRI contrast agents for high-field MRI scanners.

## 5. Conclusions

MRI has emerged as a promising imaging modality for accurate disease diagnosis. Although the population difference between the two proton spin energy states is small because of a small energy difference between them, the high content of hydrogens from water (~60 wt.% of the human body) and other sources in the human body results in a large number of hydrogen protons. This plays a critical role in producing MR signals and contrasts.

The MR signals and contrasts can be improved by accelerating the proton spin relaxation with MRI contrast agents. Commercial molecular MRI contrast agents such as Gd chelates typically have low r_1_ and r_2_ values and short blood circulation times, thus necessitating large numbers of injection doses to achieve their detection level. However, MNP-based MRI contrast agents can provide enhanced MR signals and contrast compared with those of molecular MRI contrast agents owing to their enhanced magnetic moments, high density of metals per NP, and longer blood circulation times. Moreover, MNP-based MRI contrast agents have great potential for disease therapy through drug delivery and targeting ligand conjugation on MNP surfaces. In addition, T_1_ and T_2_ dual-modal-imaging MNPs show great potential for improving disease diagnosis via complementary T_1_ and T_2_ MR images, which molecular MRI contrast agents cannot provide.

Among the MNPs, only iron oxide NPs were commercialized as T_2_ MRI contrast agents, but most of them have been withdrawn from the market. Only Resovist is currently available in a few countries. Commercial T_1_ MRI contrast agents are Gd chelates and currently hold the market for all MRI contrast agents. However, they have low sensitivity owing to their low relaxivity values and short imaging times owing to their short blood circulation times. Therefore, the development of new MRI contrast agents to overcome such shortcomings is desirable. MNPs may be the breakthrough because they have high r_1_ and r_2_ values and long blood circulation times.

## 6. Perspective

This review provides an overview of MNP-based MRI contrast agents composed of Gd, Mn, Fe, Dy, Ho, and Tb. Their high-performance as MRI contrast agents was highlighted via in vivo MRI studies. However, most of MNP-based MRI contrast agents reported in the literature are still in the development stage, with limited research carried out on in vitro and early-stage in vivo small animal studies. To increase the possibility of their safe use as high-performance MRI contrast agents in clinical trials in the future, several key issues such as toxicological effects, long-term stability, and pharmacokinetics must be addressed.

Tailoring of the particle size, morphology, composition, and surface-coating ligand is essential in achieving high-performance MNP-based MRI contrast agents. Multidisciplinary collaborative research can help advance the synthetic techniques and gain an understanding of the correlation between the fundamental physicochemical properties of MNPs and their biological behaviors in vivo and in vitro. With continuous progress in detailed scientific research on MNP-based MRI contrast agents, their use in clinical settings may become feasible in the future.

## Figures and Tables

**Table 1:** Classification of imaging modes of MNPs (++: a major application; +: a minor application).

Imagind Mode	Fe-Based MNP	Mn-Based MNP	Gd-Based MNP	Dy-Based MNP	Ho-Based MNP	Tb-Based MNP
T_1_	+	++	++			
T_2_	++	+	+	++	++	++
Dual-modal T_1_ and T_2_	+	+	+			

## Data Availability

Not applicable.
